# A phase II trial of Xeloda and oxaliplatin (XELOX) neo-adjuvant chemotherapy followed by surgery for advanced gastric cancer patients with para-aortic lymph node metastasis

**DOI:** 10.1007/s00280-014-2449-1

**Published:** 2014-04-21

**Authors:** Yan Wang, Yi-yi Yu, Wei Li, Yi Feng, Jun Hou, Yuan Ji, Yi-hong Sun, Kun-tang Shen, Zhen-bin Shen, Xin-yu Qin, Tian-shu Liu

**Affiliations:** 1 Department of Medical Oncology, Fudan University Zhongshan Hospital, 180 Fenglin Road, Shanghai, 200032 People’s Republic of China; 2 Department of Pathology, Fudan University Zhongshan Hospital, Shanghai, 200032 People’s Republic of China; 3 Department of General Surgery, Fudan University Zhongshan Hospital, Shanghai, 200032 People’s Republic of China

**Keywords:** Gastric cancer, Para-aortic lymph node, Chemotherapy

## Abstract

**Purpose:**

Gastric cancer with para-aortic lymph node (PAN) involvement is regarded as advanced disease, and only chemotherapy is recommended from the guidelines. In unresectable cases, neoadjuvant chemotherapy could prolong survival if conversion to resectability could be achieved.

**Methods:**

The study was a single-arm phase II trial. Patients who were diagnosed with gastric cancer and PAN involvement (Stations No. 16a2/16b1) were treated with capecitabine and oxaliplatin combination chemotherapy every 3 weeks for a maximum of six cycles. After every two cycles, abdominal computed tomographic scans were repeated to evaluate the response, and surgery was performed at the physician^’^s discretion in patients with sufficient tumor response, followed by chemotherapy with the same regimen to complete a total of six cycles. The primary end point was the response rate of the preoperative chemotherapy. The secondary end points were R0 resection rate, progression-free survival (PFS), overall survival (OS), and adverse events.

**Results:**

A total of 48 patients were enrolled. The response rate of the first-line chemotherapy was 49.0 %, and the clinical benefit response was 85.1 %. After a median of four cycles of chemotherapy, 28 patients received surgery (58.3 %). The median PFS and OS of all patients were 10.0 and 29.8 months, respectively. Patients in the surgery group had much longer PFS (18.1 vs. 5.6 mo, *P* = 0.001) and OS (not reached vs. 12.5 mo, *P* = 0.016) compared with those in the non-surgery group.

**Conclusions:**

For gastric cancer patients with PAN involvement, neoadjuvant chemotherapy with XELOX demonstrated a good response rate, and a sufficient R0 resection rate, with acceptable toxicities. Further study is needed to confirm the effectiveness of this regimen.

## Introduction

Despite a decrease in incidence in recent decades, gastric cancer is still one of the most common causes of cancer deaths of worldwide. In general, gastric cancer patients are diagnosed late, with high frequency of nodal involvement [1]. In advanced gastric cancer patients who underwent radical surgery, the incidence of microscopic metastasis in the para-aortic lymph node (PAN) region has been reported to range from 10 to 30 % [2]. The PAN region is defined as the terminal nodes of the stomach, and termed N3 nodes by Japanese Classification of Gastric Carcinoma [3], and as M1 nodes according to the International Union Against Cancer (UICC) tumor-node-metastasis (TNM) classification [4].

Gastric cancer with PAN metastasis is often unresectable and has a poor prognosis even after an R0 resection and extensive lymph node dissection. Furthermore, it has been reported previously that there is no survival benefit from surgery and D2 lymphadenectomy with para-aortic node dissection (D2 +PAND). The 5-year overall survival (OS) rate was found to be similar in the groups assigned to D2 lymphadenectomy alone and D2 +PAND (*P* = 0.85) [5]. The median OS of patients with PAN involvement was 13.8 months after palliative chemotherapy without surgery [6].

Some investigators have reported that potentially curable gastric cancer can be successfully treated with neoadjuvant chemotherapy [7]. Several chemotherapy regimens have been introduced in an attempt to downstage the tumor, and prolong survival following by curative resection [8, 9]. However, few previous reports have documented chemotherapy that enables the curative resection of gastric cancer with PAN involvement.

The objective of this study is to determine whether palliative chemotherapy in patients with gastric cancer and PAN involvement could make subsequent radical surgery feasible and improve OS.

## Patients and methods

### Patient selection

Patients were enrolled who had gastric or gastroesophageal adenocarcinoma proven histologically and PAN metastasis. The histological diagnosis was established by upper gastrointestinal endoscopic biopsies in all cases. The T-stage was determined by endoscopic ultrasonography (EUS), and N-stage was determined by abdominal computed tomographic (CT) scanning. Chest and pelvis CT scanning were performed to rule out distant metastases. PAN was defined as nodes in the region between the upper margin of the celiac artery and the upper border of the inferior mesenteric artery (Stations No. 16a2/16b1), and with diameters >1.0 cm by abdominal CT scanning. In addition, eligible patients were required to have an Eastern Cooperative Oncology Group performance status of 0–1.

Patients were excluded if they had peritoneal gross metastasis, lung metastasis, liver metastasis, pleural effusion, other distant metastasis, or serious uncontrolled co-morbid conditions. Patients who could not comprehend or comply with the study were also ineligible. A multidisciplinary evaluation was required before a patient’s participation in this study. All patients signed an approved written informed consent form. The protocol of this trial was approved by the institutional review board of Zhongshan Hospital, Fudan University.

### Chemotherapy schedule

Chemotherapy was given as the first-line treatment. The regimen of the chemotherapy was as follows: XELOX: capecitabine of 1,000 mg/m^2^ (orally administered twice a day on days 1–14) and oxaliplatin at 130 mg/m^2^ (on day 1, as intravenous 2 h boluses). The regimens were repeated every 3 weeks after initiation of the first cycle. Six cycles were administered during perioperative period, or treatment was stopped when disease progression was observed.

### Tumor response and toxicity criteria

After every two cycles, an abdominal CT scan was performed to evaluate the response. When PAN metastasis disappeared or shrank to <1.0 cm, surgery was considered. If surgery could not be done, chemotherapy would be continued until evidence of disease progression appeared. If operation could not be done after six cycles of chemotherapy, the patients would not have the opportunity for operation, and the treatment was stopped. The response to the treatment was evaluated according to response evaluation criteria for solid tumors (RECIST) 1.1 [10]. Pathological complete response (path CR) was defined as an absence of carcinoma cells in the primary site, and pathological partial response (path PR) was defined as <10 % residual carcinoma cells in the specimen [11]. Adverse events were assessed according to the Common Toxicity Criteria of the National Cancer Institute (NCI–CTC) 3.0 [12].

### Surgical procedure

A staging laparoscopy was performed to reveal whether there were peritoneal metastases in the patients with sufficient response to be considered for subsequent radical surgery. If there was no peritoneal metastasis, radical surgery could be done. The type of surgery performed depended on the location and extent of the primary cancer. The cancer was resected along with a gastric margin of ≥5 cm when feasible. For distal cancers, a subtotal gastrectomy was considered, and total gastrectomy or total esophagogastrectomy was performed for proximal cancers. An attempt was made to perform a D2-type nodal dissection.

### Postoperative treatment

After R0 resection, adjuvant chemotherapy with the XELOX regimen was initiated within 42 days of surgery, and six cycles were administered during perioperative period. Patients who could not undergo a radical operation, continued original chemotherapy until evidence of disease progression appeared. If patients were confirmed to have progressive disease, palliative second-line chemotherapy was administered to those patients who could tolerate it. Adjuvant radiotherapy was not administered after R0 resection. All enrolled patients were followed up regularly. Physical and blood examinations were conducted every 3 months for the first 3 years and every 6 months thereafter. An abdominal CT was performed every 6 months for the first 3 years and every year thereafter. Chest CT and upper gastrointestinal endoscopy were conducted every year.

### Study design and statistical analysis

This trial investigated the efficacy and safety of preoperative XELOX followed by surgery in gastric cancer patients with PAN involvement. The primary study end point was the response rate. Secondary end points were R0 resection rate, progression-free survival (PFS), OS, and adverse events.

PFS was measured from the date of initial treatment to the first objective documentation of disease progression or relapse. OS was measured from the start of the treatment to the date of the last follow-up or death. In this trial, the sample size was 48 cases, which provided 80 % power based on the hypothesis as the expected value of 80 %, and a threshold value of 65 % in the primary end point using one-sided testing at a 10 % significance level. All patients were followed up every 3 months. The PFS and OS were generated by the Kaplan–Meier method.

## Results

### Baseline characteristics

From November 2008 to January 2013, 48 patients were enrolled in the study. The median age was 63.5 years (range from 35 to 77 years). Other baseline characteristics of the patients are shown in Table 1.

**Table 1:** Baseline characteristics (*N* = 48)

Clinical features	*N*	%
Gender (*N*, %)		
Male	41	85.4
Female	7	14.6
Age (median year, range)	63.5 (35–77)	
Location (*N*, %)		
AEG	8	16.7
G	40	83.3
Lauren type (*N*, %)		
Intestinal type	24	50
Diffuse type	18	37.5
Mixed type	6	12.5
CEA (*N*, %)		
Normal	22	45.8
Elevated	26	54.2
Anemia		
Present (*N*, %)	37	77.1

*AEG* adenocarcinoma of esophagogastric junction, *G* gastric cancer

### Response to the chemotherapy (Table 2)

Patients received a median of four cycles of chemotherapy regimens. Forty-seven patients had responses ultimately (one did not because of acute perforation of stomach 5 days after the first regimen of chemotherapy). Two persons had a complete response (CR), 21 had partial responses (PR), 17 had stable disease (SD), and seven had progression of disease (PD). The response rate (CR + PR) was 49 % (23/47), and clinical benefit response (CP + PR + SD) was 85.1 % (40/47). The response evaluations of patients with or without surgery are shown in Table 2.

**Table 2:** Response evaluation after chemotherapy

	Number of patients (*N*)	%
Response evaluation	*n* = 47^a^	
CR	2	4.26
PR	21	44.68
SD	17	36.17
PD	7	14.89
Response of patients received surgery	*n* = 28	
CR	0	0
PR	20	71.43
SD	5	17.86
PD	2	7.14
^ a^	1	3.57
Response of patients who did not receive surgery	*n* = 20	
CR	2	10.00
PR	1	5.00
SD	12	60.00
PD	5	25.00

^a^One did not have response evaluation because of acute perforation of stomach 5 days after the first regimen of chemotherapy and had palliative surgery quickly

### Surgical findings and pathology staging (Table 3)

After response evaluation, patients whose PAN metastasis disappeared or shrank to <1.0 cm were considered for surgery. A staging laparoscopy was performed first, and three patients were diagnosed with peritoneal carcinomatosis. Then, palliative surgery was provided. At last twenty-eight patients received surgery (58.3 %, four had palliative gastrectomy, and 24 had radical gastrectomy and D2 lymphadenectomy), and 20 patients (41.7 %) did not receive operation. In the four patients who received palliative surgery, one had an acute perforation of stomach, and the other three had peritoneal metastases. Second-line treatment was administered after R2 operation. In the 20 patients who did not receive surgery after six cycles of chemotherapy, two had CR, and soon afterward stopped treatment. One had PR, but refused operation, and the original treatment was continued. Twelve had SD, and continued on first-line chemotherapy until there was evidence of disease progression. Five patients who developed progressive disease did not have second-line treatment because of poor performance status.

**Table 3:** Surgical findings after chemotherapy

	Number of patients (*N*)	%
Patients received surgery	*n* = 28	
Surgery type		
Radical surgery	24	85.71
Palliative surgery	4	14.29
Pathological response	*n* = 28	
Responders	17	60.71
Non-responders	11	39.30
pCR	3	10.70
T-stage after surgery	*n* = 28	
yT0	3	10.71
yT1	2	7.15
yT2	5	17.86
yT3	6	21.43
yT4a	9	32.14
yT4b	3	10.71
Patients with positive lymph nodes	*n* = 28	
0	8	28.57
1–2	3	10.71
3–6	4	14.29
≥7	13	46.43

In the operation group, 17 cases (60.71 %) had pathological responses, and three of them had complete pathological responses (10.7 %). The median time from surgery to discharge was 9 days (range from 7 to 15 days). Only one of the 28 patients had postoperative complications described as lung infection after surgery.

### Survival

**Fig. 1:**
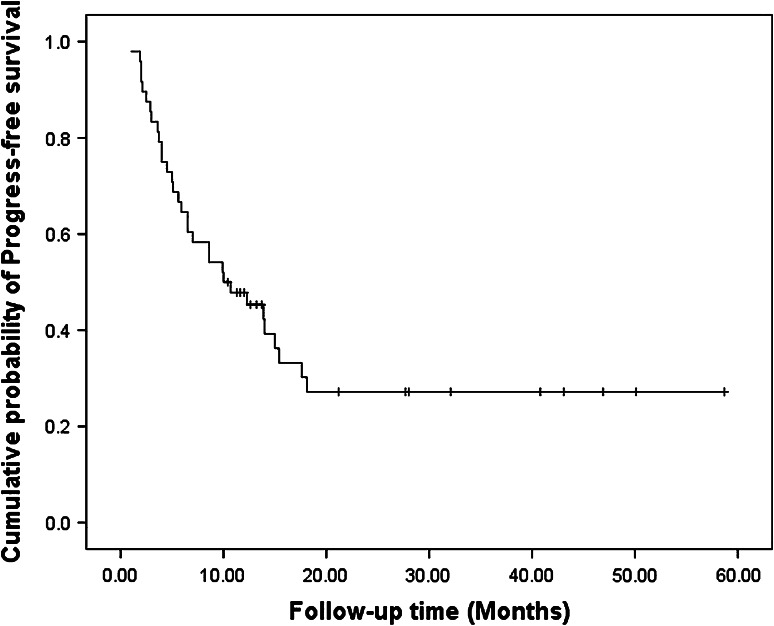
Progress free survival of all patients (*n* = 48)

**Fig. 2:**
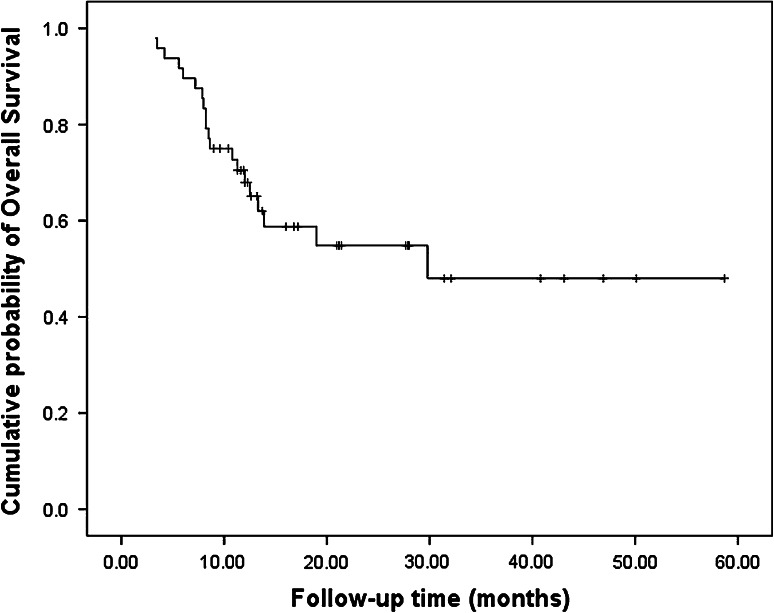
Overall survival of all patients (*n* = 48)

**Fig. 3:**
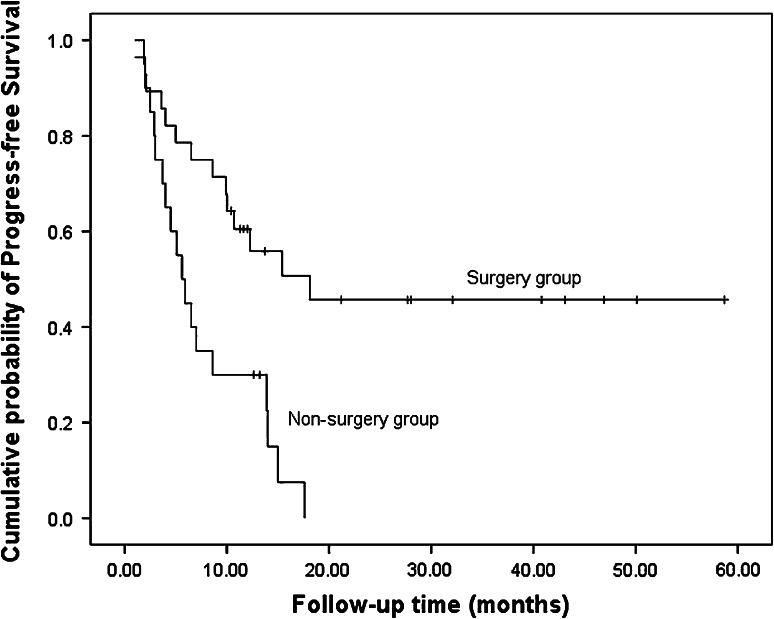
Progress free survival of surgery group and non-surgery group

**Fig. 4:**
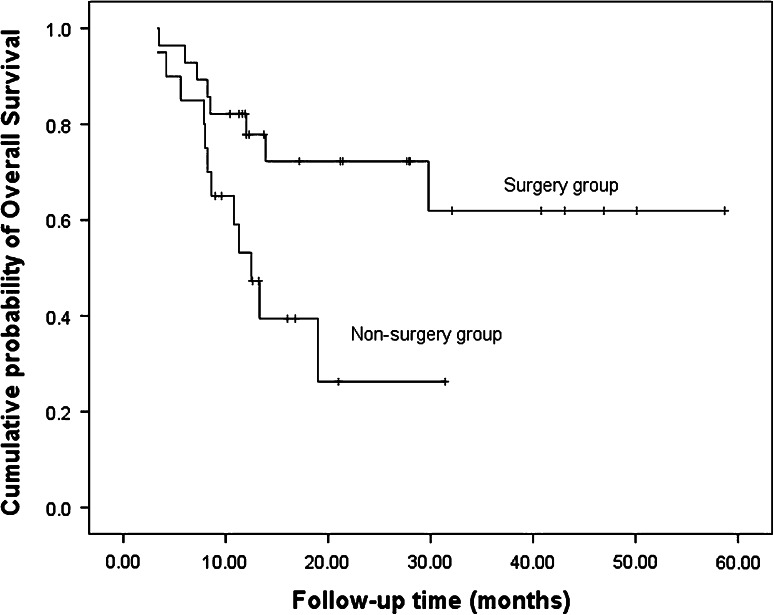
Overall survival of surgery group and non-surgery group

After a median follow-up of 12.4 months (range 3.3–58.7 mo), 20 patients died, 23 patients had disease progression, and nine patients relapsed. In the nine patients who relapsed, one had lung metastasis, one had liver metastasis, two had bone metastases, two had local recurrences, and the other three had left supraclavicular lymph node metastases. The median PFS and OS were 10.0 months (Fig. 1) and 29.8 months, respectively (Fig. 2). The 1-year PFS rate was 47.8 % and 1-year survival rate was 67.9 %. Patients in the surgery group had much longer PFS (18.1 vs. 5.6 mo, *P* = 0.001) and OS (not reached vs. 12.5 mo, *P* = 0.016) compared with the non-surgery group (Figs. 3, 4).

### Toxicity

As shown in Table 4, the most common adverse events were gastrointestinal issues and leukocytopenia. However, only one patient had grade 3 gastrointestinal toxicity and five patients had grade 3 leukopenia. No patients had grade 4 toxicities. One patient had an acute perforation of stomach 5 days after the first cycle of XELOX regimen. Only one of the 28 patients had postoperative complications described as lung infection after surgery.

**Table 4:** Toxicities of chemotherapy (*N* = 48)

Toxicities	1	2	3	4
Leukocytopenia	14	7	5	0
Thrombocytopenia	9	2	2	0
Anemia	3	1	1	0
Nausea/vomiting	8	5	1	0
Diarrhea	5	3	0	0
Hand-foot skin reaction	12	3	0	0
Hepatic dysfunction	5	0	0	0
Neuropathy	10	3	0	0

## Discussion

Patients with gastric cancers and PAN are currently considered to have distant metastatic disease and cannot be cured by surgery. However, the prognosis and clinical manifestations are somewhat different compared with other sites of metastasis. Gastric cancer patients with PAN involvement alone were found to have better survival than other advanced gastric cancer patients with multiple organ sites metastasis [6]. Therefore, it did not seem logical to simply regard gastric cancer with PAN as M1 disease and receive only palliative treatments. We have shown that the survival data in patients after palliative chemotherapy who did not receive surgery was 12.5 months which is similar to that found in other studies [6]. However, in the current study, patients who had prior chemotherapy, and subsequent radical gastrectomy, had more prolonged survival than the patients who could not have operation.

Whether surgery could be done, when surgery should be done and what kind of lymph node dissection should be done are the most demanding questions in the current study. In the current study design, we defined patients with PAN metastasis as those who had PAN >1 cm in diameter by abdominal CT scanning as an inclusion criterion. False positive findings may be caused by inflammatory lymphadenopathy. CT criteria for assessing nodal metastases are based on nodal size and shape, and the presence of central necrosis. Nodal size criteria can be used when nodes are homogeneous and clearly delineated. The generally used definition of a metastatic lymph node depends mostly on a greatest nodal diameter of more than 1 cm. With this definition, the correlation with the pathological diagnosis has been reported to be close to 80 % [13]. Therefore, when PAN metastasis disappeared or shrank to <1.0 cm, radical surgery was considered. Gastrectomy with D2 lymphadenectomy is the standard treatment for curable gastric cancer in eastern Asia, and outcomes of patients with D2 lymphadenectomy plus PAND were not significantly better than those after D2 lymphadenectomy alone in the patients with metastasis at PAN [5]. Sasako et al. reported that compared with D2 lymphadenectomy alone, treatment with D2 lymphadenectomy plus para-aortic nodal dissection did not improve survival rates in curable gastric cancer. Gastrectomy with D2 lymphadenectomy is the standard treatment for curable gastric cancer in eastern Asia. Therefore, we adopted the D2 lymphadenectomy for all patients without para-aortic lymphadenectomy. In the current study, in the seven patients who relapsed after radical operation, two had local recurrences, and five had distant metastases to lung, bone and left supraclavicular lymph node. None of them had PANs involvement, indicating that the gastric cancer patients with PAN metastasis could be cured by D2 lymphadenectomy and neoadjuvant chemotherapy. As the patients who received radical surgery had PAN metastasis disappear or shrink to <1.0 cm as determined by radiological evaluation before operation, adjuvant radiotherapy was not needed after D2 lymphadenectomy,

D2 resection is safe and effective, but the safety of D2 resection after chemotherapy had not been evaluated. In the current study, all 24 patients who received R0 resection had a D2 lymphadenectomy. The median time from surgery to discharge was 9 days, which is similar to that in patients who were not treated with neoadjuvant chemotherapy [14]. Despite one trial published by Schuhmacher et al. [15] suggesting a significantly higher risk of mortality after neoadjuvant chemotherapy, most of the randomized studies did not confirm this, as highlighted in a recent meta-analysis [16]. In the current study, only one of the 28 patients had postoperative complication which was described as a lung infection after surgery. D2 lymphadenectomy after preoperative chemotherapy is safe and effective.

An existing study showed that patients who benefitted most from neoadjuvant chemotherapy were those who achieved a complete response (pCR) with no residual microscopic tumor. However, achievement of pCR has been reported to be uncommon, occurring in only 10–15 % of patients [17], while clinical responses have been reported to range from 32 to 42 % [17–20]. In the current study, the CR rate was 10.7 %, and the clinical response rate was 49 %, which is similar to the results of other studies [17–20]. Three patients had pCR had partial clinical responses determined radiologically which indicates that CT imaging results do not always agree with histological findings.

Potentially resectable gastric cancer has been treated by several regimens of combination chemotherapy. Due to the results of the MAGIC trial [7] and Real-2 trial [21], epirubicin, cisplatin and 5-Fu (ECF) and epirubicin, cisplatin and capecitabine (EOX) are considered to be standard perioperative chemotherapy in Western countries. Among the various combination chemotherapy regimens that are currently being investigated to treat advanced gastric cancer, oxaliplatin and capecitabine (XELOX) appears to be useful and have encouraging antitumor activity [22]. Phase 2 studies have demonstrated that XELOX produces a favorable tumor response rate with a relatively mild toxicity profile [23]. The current study showed that the regimen of XELOX is safe and without severe adverse effects. The limitations of this study include small sample size and single research center data.

In conclusion, gastric cancer patients with PAN involvement can benefit from pre-surgical chemotherapy and subsequent radical surgery with D2 lymphadenectomy. Although few advanced gastric cancer patients have PAN involvement alone, the current results provide a practical treatment plan for this special group of patients. Large scale, multicenter, and randomized trials will help to further determine the best treatment strategy for these patients.
